# Novel and classical human astroviruses in stool and cerebrospinal fluid: comprehensive screening in a tertiary care hospital, Switzerland

**DOI:** 10.1038/emi.2017.71

**Published:** 2017-09-20

**Authors:** Samuel Cordey, Diem-Lan Vu, Marie-Celine Zanella, Lara Turin, Aline Mamin, Laurent Kaiser

**Affiliations:** 1 Laboratory of Virology, Infectious Diseases Service, University Hospitals of Geneva, 1211 Geneva, Switzerland; 2 University of Geneva Medical School, 1211 Geneva, Switzerland

**Keywords:** cerebrospinal fluid, classical human astroviruses, novel human astroviruses, rRT-PCR, stool, survey, tertiary care hospital

## Abstract

Classical human astroviruses (HAstV) are the third most common cause of non-bacterial acute gastroenteritis. Due to the lack of routine molecular assays, novel HAstV are underdiagnosed and the magnitude of their contribution to clinical disease remains unknown. To better understand their prevalence and the susceptible patient profile, we conducted a comprehensive screening of novel and classical HAstV in stool and cerebrospinal fluid (CSF) samples collected for clinical care in a tertiary care hospital using a specially designed rRT-PCR panel for the detection of novel (MLB1-3 and VA1-4) and classical HAstV. Of the 654 stool samples, 20 were positive for HAstV, and the novel (*n*=10; 3 MLB1, 4 MLB2; 3 VA2) and classical (*n*=10) serotypes were equally prevalent. None of the 105 CSF samples were positive. Investigating the patient profile, we found a higher prevalence (*P*=0.0002) of both novel and classical HAstV in pediatric stool samples (3.4% and 3%, respectively) compared with adult stool samples (0.5% and 0.7%, respectively). Furthermore, all novel and classical HAstV-positive pediatric subjects were ≤four years old, demonstrating similar susceptible populations. Forty-five percent of positive patients were immunocompromised (novel: 40%, classical: 50%). A comparison of novel and classical HAstV-positive cases showed a lower viral load for novel HAstV (*P*=0.0007) with significantly more upper respiratory symptoms (70% of subjects; *P*=0.02); this observation may suggest a unique pathogenic pathway. This study confirms the clinical and epidemiological relevance of novel HAstV and identifies a target population in which routine screening may yield clinically valuable information.

## INTRODUCTION

Human astroviruses (HAstV) are non-enveloped, single-stranded RNA viruses of the *Astroviridae* family. Classical HAstV are a major cause of viral diarrhea^1, 2^ and affect mainly children,^3, 4^ the elderly^5, 6, 7^ and immunocompromised^8, 9^ subjects. In the immunocompromised population, severe digestive symptoms of long duration and potentially fatal disseminated infections have been reported.^8, 9, 10^ Classical HAstV are classified into eight distinct serotypes belonging to the *Mamastrovirus* (MAstV) *1* species. HAstV-1 is the most commonly detected serotype^11^ with a seroprevalence of up to 90% in children by the age of 5 years.^12, 13^

Recently, novel HAstV (MLB and VA) phylogenetically distant from classical HAstV have been identified in humans.^14^ Although the correlation between novel HAstV and diarrhea remains to be clarified,^15, 16, 17, 18^ they have been reported as the causal agents for meningitis and encephalitis, mainly in immunocompromised patients.^19, 20, 21, 22, 23, 24^ Furthermore, we recently detected novel HAstV VA1 in a nasopharyngeal swab specimen from a 13-month-old patient suffering from an acute respiratory disease of unknown etiology.^25^ However, the available studies offer limited information on the epidemiology and respective distribution of the novel HAstV known to infect humans. The current routine molecular assays only target the classical HAstV serotypes 1–8, and in the absence of appropriate diagnostic tools, novel HAstV remain largely underdiagnosed.

This study aims to investigate the epidemiological and clinical relevance of novel HAstV and the pediatric and adult patient profiles that they are most likely to affect. To this end, we designed a specific rRT-PCR panel to perform systematic and standardized screening for both novel and classical HAstV. Given the known gastrointestinal replication of classical HAstV and the increasing number of reports linking HAstV to unusual cases of meningoencephalitis, systematic and standardized screening of classical and novel HAstV was conducted on stool and cerebrospinal fluid (CSF) samples collected as part of routine clinical care and sent to our virology laboratory. HAstV prevalence and demographic, epidemiological and clinical data were analyzed systematically.

## MATERIALS AND METHODS

### Ethics statement

This study was approved by the research ethics committee of the University Hospitals of Geneva (project # 2016-01096).

### Study design

Stool specimens were collected between 1 November 2014 and 31 October 2015 from pediatric (≤15-year-old) and adult patients (with or without diarrheal disorders) admitted or attending (in- and outpatient) the University Hospitals of Geneva (Geneva, Switzerland). These samples were obtained when viral screening was requested by the physician in charge and were then tested for the presence of classical and novel HAstV. In the event that several samples were collected from the same subject within a period of less than 30 days, a single sample (the earliest) was selected for analysis. Cerebrospinal fluid samples with a total leukocyte count of >5 cells/μL collected between 1 May 2015 and 31 July 2016 that were sent to the laboratory of virology for viral screening were included in the study when sufficient leftover volume was available. Stool and CSF samples were stored at −80 °C before analysis.

### Design and validation of the specific rRT-PCR panel

A genetic alignment analysis was performed, comparing all classical and novel HAstV sequences available in GenBank up to August 2015. Based on this alignment, specific primers and probes were designed using Primer Express software v3.0 (Applied Biosystems, Rotkreuz, Switzerland). The nucleotide sequences of primers and probes are shown in Supplementary Table S1. For each rRT-PCR assay, standard curves and the lower limit of detection were assessed using 10-fold serial dilutions of specific RNA oligonucleotides (Microsynth, Balgach, Switzerland) consisting of the target sequences (Supplementary Table S1). The standard curve for the MLB1, MLB2, MLB2-3, VA1, VA2, VA3, VA4 and Hast rRT-PCR assays were obtained using RNA oligonucleotides corresponding to the GenBank accession numbers NC_011400, KT224358, NC_019028, NC_013060, GQ502193, JX857868, JX857869 and FJ755402 (HAstV-1), respectively.

### Screening of novel and classical HAstV

Due to the diverse composition of stool, standardization of the re-suspension process is typically challenging. The method of re-suspension used in this study was optimized to ensure minimal impact on the viral load measurement. Thus, the re-suspension process is not considered to significantly influence the viral loads measured in this study. Briefly, 10 μL of solid stool (using a 10-μL loop) or 500 μL of liquid stool were resuspended in 5 mL of 1 × phosphate-buffered saline. After vortexing, an aliquot of 1 mL was stored at −70 °C.

The viral genome was extracted individually from 380 μL of CSF and 760 μL of phosphate-buffered saline-resuspended stool samples using the NucliSENS easyMAG (bioMérieux, Geneva, Switzerland) nucleic acid kit. As an internal control, 20 and 40 μL of standardized canine distemper virus were added to each CSF and stool sample, respectively, before extraction.^26^ Elutions were performed in 50 and 100 μL, respectively, for the CSF and stool samples. Overall, eight different rRT-PCR assays were needed for the intended screening. The MLB1, MLB2, MLB2-3, VA1, VA2, VA3, VA4 and an updated version of the Hast^27^ rRT-PCRs were performed using the one-step QuantiTect Probe RT-PCR Kit (Qiagen, Hombrechtikon, Switzerland) in a StepOne Plus instrument (Applied Biosystems) under the following cycling conditions: 50 °C for 30 min, 95 °C for 15 min, 45 cycles of 15 s at 94 °C and 1 min at 55 °C. For each rRT-PCR assay, RNA concentrations (RNA copies/mL of resuspended stool) were estimated by reporting Ct values on standard curves previously obtained from serial dilutions of specific RNA oligonucleotides containing the target sequences.

### Classical HAstV typing

Extracted RNA from classical HAstV-positive stool samples were reverse transcribed with random hexamers (Roche, Indianapolis, IN, USA) using reverse transcriptase SuperScript II (Invitrogen, Carlsbad, CA, USA). A 449-bp region of the capsid protein was amplified using the primers Mon269 and Mon270 as previously described.^28^ Sequences were analyzed with the Geneious version 6.1.8 software platform and blasted in GenBank.

### Data collection

A retrospective review of the medical records of the 20 patients with positive stool samples was performed to collect patient characteristics and clinical data.

### Statistical analysis

Statistics were performed using Stata (StataCorp. 2015, College Station, TX, USA). For continuous variables, differences between groups were tested using the Mann–Whitney *U*-test. For categorical variables, differences between groups were tested using *χ*^2^ or Fisher’s exact test. A two-sided *P*-value of <0.05 was considered significant.

## RESULTS

### Prevalence of novel and classical HAstV in stool and CSF samples

**Figure 1:**
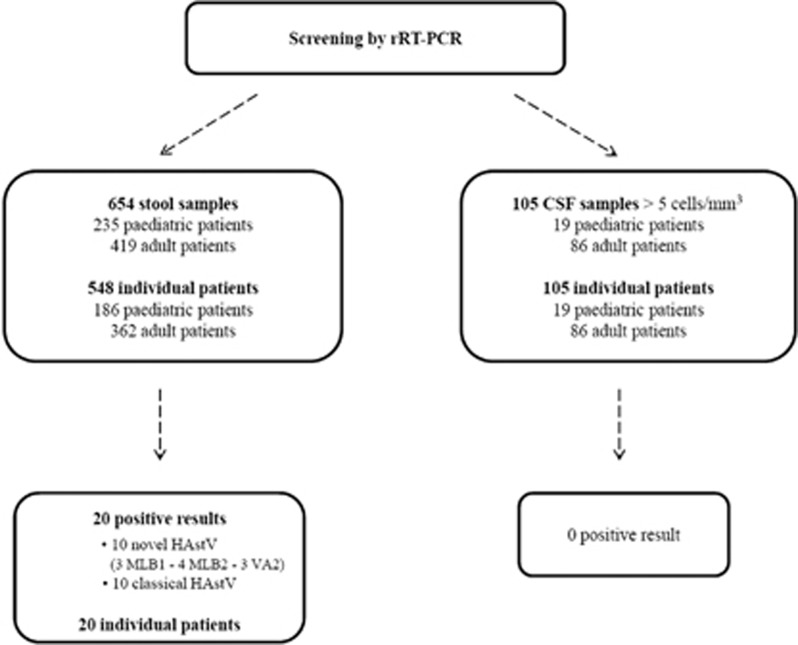
Study flowchart using eight rRT-PCRs to detect human astroviruses in stool and cerebrospinal (CSF) samples sent for viral screening in the laboratory of virology, University Hospitals of Geneva, 2015–2016.

**Figure 2:**
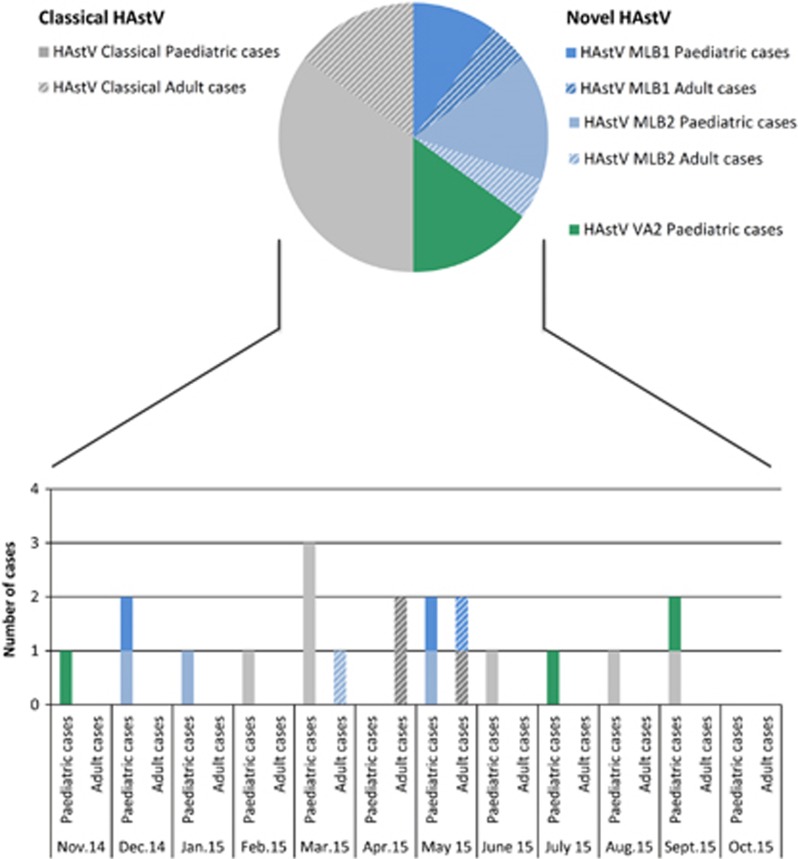
Positive human astroviruses in stool samples. Top: Pie chart showing the prevalence and composition of classical and novel astroviruses among HAstV-positive patients. Bottom: histogram of the temporal distribution of cases. human astrovirus, HAstV.

A total of 654 stool specimens collected from 548 individual patients (186 pediatric and 362 adult cases) were tested by rRT-PCR (Figure 1 and Supplementary Figure S1). The overall median age of the 548 screened patients was 52 years old (range, 0–100). The median age of the 186 pediatric patients was 1 year old (range, 0–15). Twenty of the 654 analyzed stool samples were positive for HAstV (3.1% overall prevalence) with 10 classical HAstV and 10 novel HAstV (Figure 2 and Table 1). Fifteen of the 235 pediatric samples (6.4%) and five of the 419 adult samples (1.2%) were positive for HAstV. A comparative analysis of positive stool samples in the pediatric and adult populations showed a significantly higher prevalence of all HAstV among pediatric patients (*P*=0.0002) with a risk ratio of 5.4 (95% CI (1.9, 14.5)). Similarly, when analyzing novel and classical HAstV separately, the prevalence of either a novel or a classical HAstV was also significantly higher among pediatric patients (*P*=0.006 and *P*=0.04, respectively) with a risk ratio of 7.1 (95% CI (1.5, 33.3)) and 4.2 (95% CI (1.1, 15.9), respectively). Classical HAstV were detected in 1.5% of all stool samples (pediatric, 3% adult, 0.7%). Novel HAstV were also detected in 1.5% of all stool samples (pediatric, 3.4% adult, 0.5%). Regarding pediatric HAstV-positive subjects, novel HAstV were detected in neonates (one case), infants (that is, 1 month–2 years; three cases) and children (four cases), whereas classical HAstV were restricted to infants (four cases) and children (three cases) (Table 1).

Screening revealed the presence of 3 MLB1, 4 MLB2, 3 VA2 and 10 classical HAstV (Table 1 and Figure 2), resulting in a prevalence of 0.46%, 0.61%, 0.46% and 1.53%, respectively. No cross-reaction was observed between the different rRT-PCR assays, demonstrating their excellent respective specificities. HAstV-1 was the most frequent serotype detected among classical HAstV (Table 1). These results show that novel HAstV were detected with the same frequency as classical HAstV in stool samples sent for routine screening.

**Figure 3:**
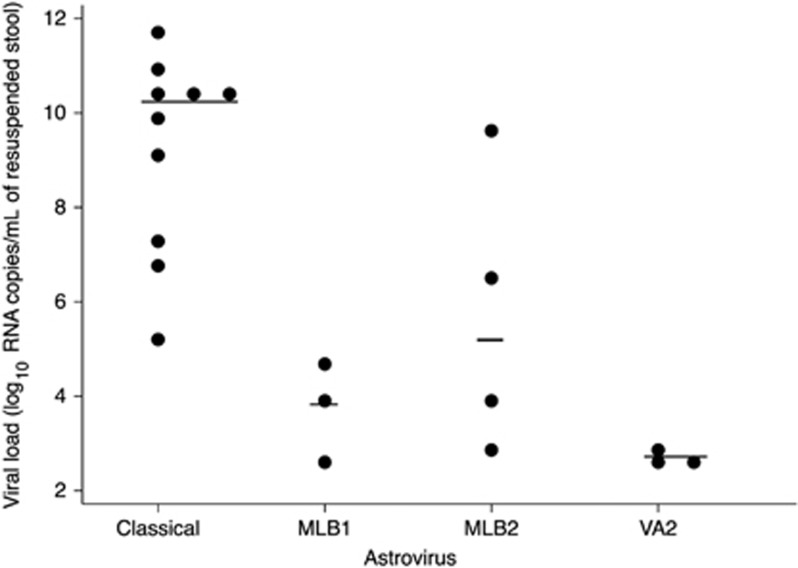
Classical and novel human astrovirus viral load in stool samples. The horizontal lines denote median log values. ***P<0.001. human astrovirus, HAstV.

The viral load was estimated for each positive case (Table 1 and Figure 3) and ranged from 7.1 × E2 to 3.3 × E9 for MLB2, 3.3 × E2 to 5.5 × E4 for MLB1, 3.4 × E2 to 8.4 × E2 for VA2 and 2.1 × E5 to 6.0 × E11 for classical HAstV. Overall, the viral load was significantly lower (*P*=0.0007) in novel HAstV-positive (MLB1, MLB2 and VA2) than in classical HAstV-positive samples (Table 2). This latter observation did not result from different time intervals between the symptom onsets and the collection of samples (*P*=0.95). Due to the small number of positive samples, a statistical analysis could not be performed to compare viral loads between the different groups of novel HAstV. The mean viral load of all HAstV-positive samples was similar between adults and children (*P*=0.137) and when novel and classical HAstV were considered separately (*P*=0.433 and *P*=0.253, respectively). The distribution of novel and classical HAstV-positive cases was uniform throughout the year, irrespective of the studied population (Figure 2).

A total of 105 CSF samples collected from 105 individual patients (19 pediatric; 86 adult) were tested. Among these, none were positive (Figure 1).

### Clinical features in HAstV-positive cases

Among the 20 patients whose samples were positive, the median age was 3.3 years old (range, 23 days–53 years old). The median age of pediatric patients was 1 year old (range, 23 days–4 years old) with a male-to-female ratio of 0.88:1. The median age of adult patients was 40 years old (range, 30–53 years old) with a male-to-female ratio of 0.7:1. Nine (45%) of the 20 patients were immunocompromised (Table 1). Six patients were solid organ transplant recipients and three were allogeneic stem cell transplant recipients. The median time between transplantation and HAstV detection was 617 days (range, 6–1139). Of the 20 positive patient samples, 13 were collected from outpatients. Seven patients were hospitalized at the time of stool sample collection.

Fever was reported in seven of the 20 HAstV-positive patients (Table 1). Upper respiratory manifestations were reported in 8 of 20 patients, with significantly more patients in the novel HAstV group (*n*=7, 70%) compared to the classical HAstV group (*n*=1, 10%) (*P*=0.02). Upper respiratory manifestations were reported in all patients (*n*=3) with HAstV-VA2-positive stool samples (Tables 1 and 2). The mean time between upper respiratory manifestations and HAstV detection was 11.5±5.9 days.

Routine diagnostic rRT-PCR was performed to detect enteric viruses (norovirus,^29^ rotavirus,^30^ enterovirus^31^ and adenovirus (adapted from ref. 32)) in the 20 HAstV-positive cases. This screening was performed on the same stool samples used in HAstV analysis, and enteric viruses were detected in three patients. One was a liver transplant recipient who tested positive for rotavirus (patient no. 3; Table 1) while the remaining two were immunocompetent patients with enterovirus (patient nos. 9 and 12; Table 1).

## DISCUSSION

This study describes a unique survey of classical and novel HAstV among pediatric and adult patients in a tertiary care hospital using a panel of specifically designed rRT-PCR assays.

Our investigation showed that novel and classical HAstV were detected with the same frequency in stool samples obtained from routine viral screening in a tertiary care hospital. Similar to other studies, HAstV-1 was the most frequent serotype detected among classical HAstV.^18^ Our systematic approach over a 1-year period revealed that, of the novel HAstV, only MLB1, MLB2 and VA2 were consistently detected. This finding is in agreement with previous observations in which MLB1, MLB2, MLB3 and VA2 were the novel HAstV most frequently detected in studies among patients with diarrhea.^14^ However, in our study, screening did not reveal the presence of MLB3, VA1, VA3 or VA4; this observation cannot be attributed to a lower sensitivity of rRT-PCR assays (Supplementary Table S1). While these observations might be expected for VA1, VA3 and VA4, the absence of MLB3 may result from differences in prevalence, depending on factors such as geographical location, climate or year of the study.^18^ An analysis of the monthly distribution of HAstV-positive cases revealed a uniform distribution of both the number of stool samples tested and the positivity rate throughout the study period (Supplementary Figure S2). Given the small number of cases, it is not possible to form conclusions on seasonality trends.

Investigating the patient profile of HAstV-positive patients revealed a clearly susceptible population. A significantly higher prevalence of novel and classical HAstV-positive samples was observed among pediatric patients compared to adults. All novel and classical HAstV-positive pediatric subjects were ≤four years old. Almost half of all HAstV-positive cases were immunocompromised patients. Children and immunocompromised patients have been previously described as key susceptible populations for classical HAstV. Our results corroborate this finding for classical HAstV and further show this trend to be mirrored in novel HAstV (Table 1). As viremia and dissemination to other organs have been described for HAstV,^10, 11, 33^ all available additional samples collected for routine care before and/or after the positive samples (respiratory, stool, serum, plasma, CSF and/or biopsy samples) were tested retrospectively for the 20 HAstV-positive cases. However, none of these additional samples returned a positive result (data not shown), thus suggesting that HAstV in these 20 patients were present as a transient infection, not a chronic one. The absence of positive CSF samples for novel and classical HAstV is consistent with other studies. Indeed, only three cases of novel HAstV-associated encephalitis with HAstV-positive CSF have been described.^19, 20, 24^ The remaining published cases of novel HAstV-associated encephalitis were detected in brain biopsies.^14^ Of note, of the 105 patients screened in this study, 89 were immunocompetent; this could explain the absence of novel HAstV detection since most of the novel HAstV-associated meningoencephalitis cases were reported in immunocompromised patients. Taken together, HAstV-associated meningoencephalitis cases are rare events, and CSF screening should be considered in specific patients.

A review of medical records revealed that up to 70% of novel HAstV-positive subjects presented upper respiratory manifestations a few days before stool sampling vs only 10% of classical HAstV-positive cases. Although this observation needs to be confirmed with larger and specific prospective studies, it reflects the observation of a recently published case in which HAstV VA1 was detected in a nasopharyngeal specimen of a 13-month-old Tanzanian child with acute respiratory symptoms.^25^ Furthermore, the mean viral load of novel HAstV was significantly lower than that seen in classical HAstV-positive cases even though the sensitivity of the Hast rRT-PCR assay appears to be the lowest (Supplementary Table S1). However, this observation should be considered with caution as the difference is probably biased by the very low viral loads detected in the three VA2-positive subjects (Table 1). Taken together, and similar to observations for the different adenovirus and enterovirus genotypes, our study offers potential evidence for a divergence in the pathogenic pathways of classical and novel HAstV. Although all novel HAstV-positive cases presented gastroenteritis symptoms (Table 1), it could be postulated that some novel HAstV have a tropism for upper respiratory epithelia, eventually leading to upper respiratory manifestations and followed by subsequent gastroenteritis-like symptoms. However, it remains unknown whether the presence of gastroenteritis-like symptoms reflects a possible viral replication in enteric cells (as suggested by the high MLB2 viral loads detected in patient nos. 5 and 7; Table 1) or if these symptoms are indirect or confounding phenomena. Indeed, recent data showed that the TAstV-2 capsid protein could act as an enterotoxin and that its oral administration was sufficient to induce diarrhea in turkey poults.^34^ Comprehensive HAstV screening in routine respiratory samples is needed to better investigate the potentially divergent pathogenic pathways of novel and classical HAstV.

Similar to previous findings, the present study revealed the co-detection of novel or classical HAstV with other pathogens (Table 1).^16, 35^ Despite the well-known association between classical HAstV and gastroenteritis, it remains to be determined whether novel HAstV have the potential to act as unique pathogenic agents or if they are simply bystanders of other enteric agents.

To our knowledge, this study represents the first epidemiologic description of novel HAstV in a tertiary care hospital based on pediatric and adult sample screening. Since this study included all stool samples collected from pediatric and adult patients (with or without diarrheal disorders) for which a viral screening was requested by physicians, the observed prevalence of novel and classical HAstV is likely to be underestimated. Nevertheless, although our results are in line with previously published prevalence reports in patients <5 years old with moderate-severe diarrhea in Kenya and The Gambia,^17^ the observed prevalence of novel HAstV in the pediatric population (3.4%) was far higher than what has been previously reported in most studies. This result is true either in symptomatically selected patients (that is, those with diarrhea or acute flaccid paralysis) or specifically among hospitalized children.^14^ Given the retrospective nature of this study, patient characteristics were also based on a retrospective analysis of medical records. This analysis method is particularly important for clinical characteristics, such as respiratory manifestations. Finally, the medical records of HAstV-negative patients were not reviewed in this study. However, further investigations comparing the clinical features between HAstV-positive and -negative patients would be valuable.

In conclusion, this study confirms the clinical and epidemiological relevance of novel HAstV and may help advance the future implementation of novel HAstV molecular assays, such as rRT-PCR. We also identify the target population, namely, immunocompromised and pediatric patients, in which routine screening may yield clinically valuable information.

## Figures and Tables

**Table 1:** Clinical cases of classical and novel HAstV detection in stool samples

**Patient characteristics**					**HAstV characteristics**		**Enteric virus stool screening**	**Clinical manifestations**				
**Patient**	**Sex**	**Age**	**Immunodeficiency, etiology**	**Time between transplantation and HAstV detection**	**HAstV detected**	**Viral load (RNA copies/mL of resuspended stool)**	**Virus specie**	**Fever**	**Digestive symptoms, time between symptoms and HAstV detection**	**Type of digestive symptoms**	**Respiratory symptoms, time between symptoms and HAstV detection**	**Type of respiratory symptoms**
1	M	3.1 years	No		HAstV-MLB1	5.5 × E4	Neg.	No	Yes, 2 days	Vomiting, diarrhea	Yes, 15 days	Cough
2	F	3.4 years	No		HAstV-MLB1	6.1 × E3	Neg.	Yes	Yes, 4 days	Diarrhea, abdominal pain	No	
3	F	51.0 years	Yes, liver transplant recipient	26.4 months	HAstV-MLB1	3.3 × E2	Rotavirus	No	Yes, 1 day	Vomiting, diarrhea, abdominal pain	Yes, 1 day	Cough
4	M	4.2 years	Yes, liver transplant recipient	37.5 months	HAstV-MLB2	7.1 × E2	Neg.	No	Yes, 1 day	Diarrhea	Yes, 13 days	Cough, rhinorrhea
5	F	9.8 months	No		HAstV-MLB2	2.9 × E6	Neg.	No	Yes, 14 days	Diarrhea	Yes	Cough
6	M	2.4 years	Yes, liver transplant recipient	15.4 months	HAstV-MLB2	7.2 × E3	Neg.	No	Yes, 21 days	Diarrhea	Yes, 10 days	Odynophagia
7	M	38.2 years	Yes, allogeneic stem cell transplant recipient	4.7 months	HAstV-MLB2	3.3 × E9	Neg.	No	Yes, 3 days	Vomiting	No	
8	F	1.4 months	No		HAstV-VA2	4.8 × E2	Neg.	Yes	Yes, 4 days	Diarrhea	Yes, 10 days	Rhinorrhea
9	F	23 days	No		HAstV-VA2	8.4 × E2	Enterovirus	Yes	Yes, 2 days	Diarrhea	No	
10	M	5.1 months	No		HAstV-VA2	3.4 × E2	Neg.	No	Yes, 1 day	Diarrhea	Yes, 16 days	Cough, rhinorrhea
11	F	9.6 months	Yes, liver transplant recipient	6 days	Classical HAstV undet.	2.3 × E7	Neg.	Yes	Yes, 1 day	Diarrhea	No	
12	M	4.2 years	No		Classical HAstV-8	2.0 × E10	Enterovirus	Yes	Yes, 2 days	Diarrhea	No	
13	M	4.8 years	Yes, kidney transplant recipient	37.9 months	Classical HAstV undet.	2.1 × E5	Neg.	No	Yes, 4 days	Diarrhea	Yes, 7 days	Cough
14	M	1.4 years	No		Classical HAstV-1	2.4 × E10	Neg.	No	Yes, >30 days	Diarrhea	No	
15	F	9.9 months	No		Classical HAstV-2	1.0 × E11	Neg.	No	Yes, 1 day	Vomiting, diarrhea	No	
16	F	6.4 months	No		Classical HAstV-1	5.3 × E6	Neg.	Yes	Yes, 14 days	Vomiting, diarrhea	No	
17	F	3.4 years	Yes, liver transplant recipient	20.6 months	Classical HAstV-4	1.6 × E9	Neg.	Yes	Yes, 2 days	Vomiting, diarrohea	No	
18	M	40.2 years	Yes, allogeneic stem cell transplant recipient	25.4 months	Classical HAstV-1	7.5 × E9	Neg.	No	Yes, 5 days	Diarrhea	No	
19	F	30.4 years	No		Classical HAstV-3	2.0 × E10	Neg.	No	Yes, 1 day	Vomiting, diarrhea	No	
20	F	53.0 years	Yes, allogeneic stem cell transplant recipient	11.3 months	Classical HAstV-5	6.0 × E11	Neg.	NA	NA	NA	NA	NA

Abbreviations: human astrovirus, HAstV; not available, NA; negative, neg; undetermined, undet.

**Table 2:** HAstV-positive stool samples: overall and stratified (classical and novel) HAstV patient characteristics

	**All HAstV** ***n*****=20**	**Classical HAstV** ***n*****=10**	**Novel HAstV** ***n*****=10**	***P*** **value**^a^
*Patient characteristics*				
Male sex – no. (%)	9 (45)	4 (40)	5 (50)	
Age of pediatric and adult patients (yo)				0.3258
Median (range)	3.3 (0.06–53.0)	3.8 (0.5–53.0)	2.8 (0.06–51.0)	
Mean±sd	12.2±18.6	14.0±19.6	10.4±18.4	
Pediatric patients, no. (%)	15 (75)	7 (70)	8 (80)	1.000
Age of pediatric patients, months				0.3545
Median (range)	16.7 (0.8–57.7)	16.7 (6.4–57.7)	19.2 (0.8–50.0)	
Mean±sd	24.4±20.1	27.3±21.6	21.8±19.8	
Immunocompromised – no. (%)	9 (45)	5 (50)	4 (40)	1.000
Fever – no. (%)	7 (36.8)^b^	4 (44.4)^c^	3 (30)	
With respiratory symptoms – no. (%)	8 (42.1)^b^	1 (11.1)^c^	7 (70)	0.020
Time interval between respiratory symptoms and HAstV detection (days)				
Median (range)	11.5 (1–20)	7	13 (1–20)	
Mean±sd	11.5±5.9	7	12.1±6.0	
Time interval between transplantation and HAstV detection (days)				
Median (range)	617 (6–1139)	617 (6–1139)	626.5 (142–1126)	
Mean±sd	598.1±399.9	572.4±428.3	630.3±423.6	

*HAstV*				
Viral load (RNA copies/mL of resuspended stool)				0.0007
Median (range)	4.1 × E6 (3.4 × E2–6.0 × E11)	1.4 × E10 (2.1 × E5–6.0 × E11)	3.5 × E3 (3.4 × E2–3.3 × E9)	
Mean±sd	3.9 × E10±1.3E × 11	7.7 × E10±1.9 × E11	3.3 × E8±1.0 × E9	

Abbreviations: human astrovirus, HAstV; standard deviation, sd; years old, yo.

a *P*-values are for the comparison between the classical and novel HAstV-positive stool sample groups.

b Percentage calculated based on 19 patients (1 missing data, Table 1).

c Percentage calculated based on 9 patients (1 missing data, Table 1).
