# Supplementary figures and images for: Novel and classical human astroviruses in stool and cerebrospinal fluid: comprehensive screening in a tertiary care hospital, Switzerland
Source: Emerg Microbes Infect. 2017 Sep 20;6(9):e84–. doi: 10.1038/emi.2017.71 (PMC5625321; doi:10.1038/emi.2017.71)

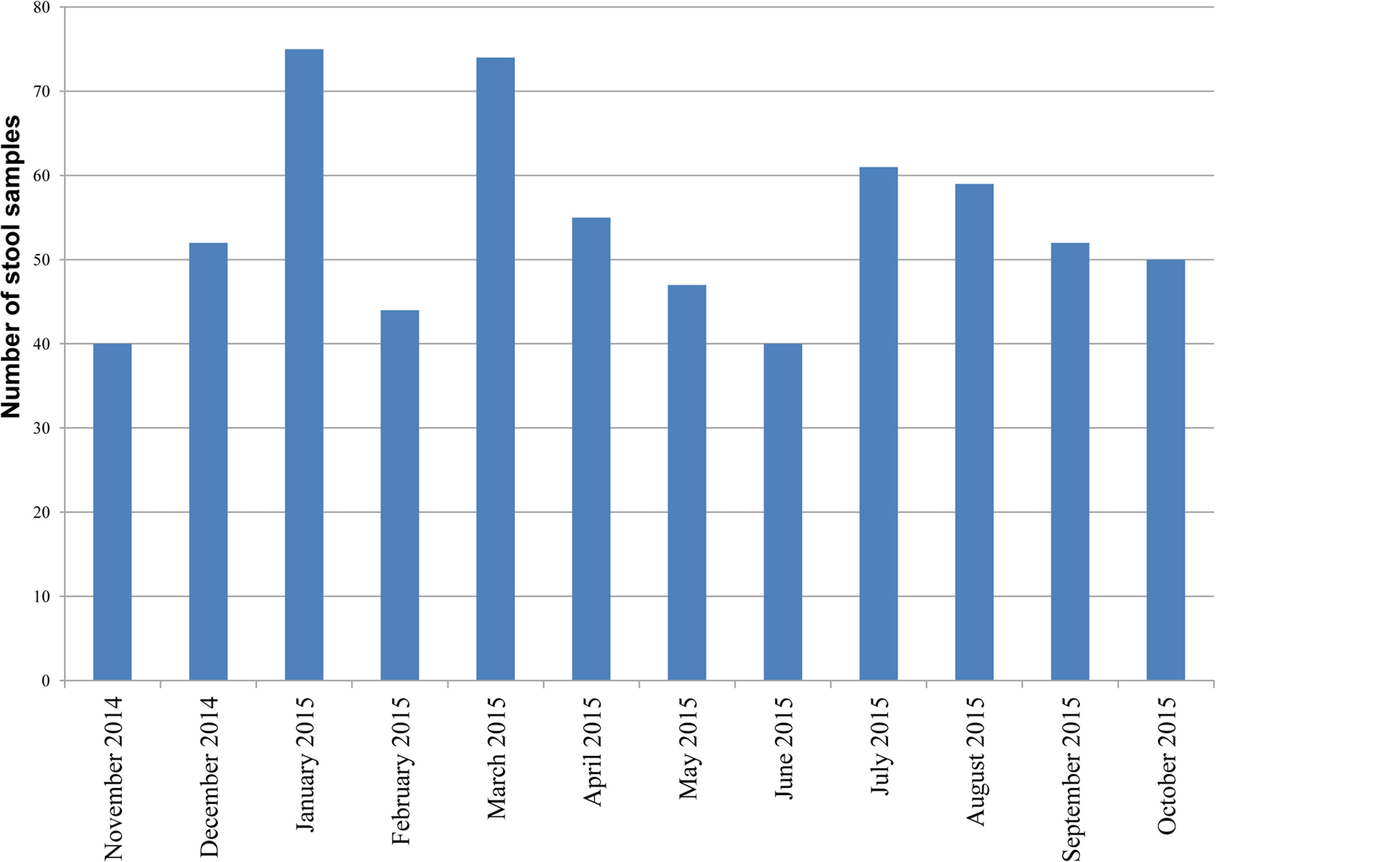

Supplement: Supplementary Figure S2 [file emi201771x2.tif]
