# Supplementary material for: Novel and classical human astroviruses in stool and cerebrospinal fluid: comprehensive screening in a tertiary care hospital, Switzerland
Source: Emerg Microbes Infect. 2017 Sep 20;6(9):e84–. doi: 10.1038/emi.2017.71 (PMC5625321; doi:10.1038/emi.2017.71)
Supplement: Supplementary Table S1 [file emi201771x3.docx]

| **Assay** | **Viruses Detected** | **Target Region (ORF)** | **Amplicon size**  (nucleotids) | **Sequence (5' to 3')** | | | **Final [uM]**  **Fwd/Rev/Probe** | **Standard curve**  **(10-fold serial dilutions)** | | | **Reference** |
| --- | --- | --- | --- | --- | --- | --- | --- | --- | --- | --- | --- |
|  |  |  |  | **Fwd primer** | **Probe** | **Rev Primer** |  | **Lower limit of detection (100%) (**RNA copies/reaction) | **Slope** (ln(x)) | **R^2^** |  |
| **MLB1** | MLB1 | ORF2 | 68 | GGTCTTGGAGCYCGAATTC | FAM - TAGRGTTGGTTCAAATCT – MGBNFQ | CGCTGTTTAATGCGCCAAA | 0.6/0.6/0.25 | 5 | -1.47 | 0.99 | This study |
| **MLB2** | MLB2 | ORF2 | 68 | ACAACTGGCCCTACATTGAATTC | FAM - TCGGGTCTTGGCGCGCGAT –TAMRA | CCGACACGCACATCTCGAT | 0.9/0.9/0.2 | 50 | -1.46 | 1 | (33) |
| **MLB2-3** | MLB3; low sensitivity for MLB2 | ORF1b | 71 | CCGAGCTCTTAGTGATGCTAGCT | FAM - CGCTTCACTCGGAGAC – MGBNFQ | CACCCCTCCAAATGTACTCCAA | 0.6/0.6/0.2 | 50 | -1.43 | 1 | This study |
| **VA1** | VA1 / HMO-C / SG / PS / UK1 | ORF2 | 66 | CCATCAGCAGTTACYGGGTCTGT | FAM - TTTCCGCATATCCC – MGBNFQ | CGTGGCTCCAGGTGAYTGT | 0.6/0.6/0.2 | 50 | -1.38 | 0.99 | This study |
| **VA2** | VA2 / HMO-A | ORF2 | 67 | CAGGGCCTGAATTACAAATTTCA | FAM - CATTTATGCATCCTGCTTT – MGBNFQ | GTGCCATCATTTGGCTCTTTC | 0.9/0.9/0.25 | 50 | -1.45 | 1 | This study |
| **VA3** | VA3 / HMO-B | ORF1b | 67 | TTCCAGGCATTTGAGTTTGCT | FAM - TTGAATCCGGATAAAAC – MGBNFQ | CCCATCCTTCTCTCAGTTCATCA | 0.6/0.6/0.25 | 50 | -1.47 | 1 | This study |
| **VA4** | VA4 | ORF2 | 62 | GATCCATGTATCGTGCATCGTT | FAM - AACCTTACACAGTCCCCGG – MGBNFQ | GCCCCCCCAAGATGTTG | 0.9/0.9/0.2 | 50 | -1.46 | 1 | This study |
| **Hast** | Classical serotypes 1-8 | ORF1b | 64 | CCAGACTCACWGAAGAGCAAC | FAM-CATCGCATTTGGA- MGBNFQ | TAGCCATCRCACTYCTTTGG | 0.6/0.6/0.2 | 500 | -1.51 | 0.96 | adapted from (27) |
